# Diagnostic imaging in a patient with an isolated blunt traumatic gallbladder injury

**DOI:** 10.1016/j.radcr.2021.06.036

**Published:** 2021-07-09

**Authors:** Hong Duc Pham, Tran Canh Nguyen, Quang Huy Huynh

**Affiliations:** a Radiology Department, Ha Noi Medical University, Ha Noi, Vietnam; b Radiology Department, Saint-Paul Hospital, Ha Noi, Vietnam; c Radiology Department, Pham Ngoc Thach University of Medicine, 2 Duong Quang Trung street, District 10, Ho Chi Minh city 700000, Vietnam; d Radiology Department, Trung Vuong Hospital, Ho Chi Minh city, Vietnam

**Keywords:** Isolated gallbladder injury, Blunt abdominal trauma, Radiographic findings

## Abstract

Isolated gallbladder injuries are very uncommon in blunt abdominal trauma due to its small size. Further, they are well protected by the surrounding liver, omentum, and the rib cage. A case of traumatic gallbladder injury in a 47-year-old man with progressive right hypochondrial pain is presented. The gallbladder injury was caused due to a blunt abdominal trauma after a motor vehicle accident. The patient had a history of chronic alcoholism and narcotics abuse. The patient was also human immunodeficiency virus-positive and was on stable treatment for tuberculosis. A diagnosis of gallbladder contusion with intramural dissection was made after an ultrasound and computed tomography scan. However, the patient refused surgery and thus, an ultrasound-guided percutaneous transhepatic drainage of the gallbladder was performed as a temporary treatment. Subsequently, a successful cholecystectomy was performed. Isolated traumatic gallbladder injury has been reviewed due to the rarity of this condition and the diagnostic challenges it poses.

## Introduction

Isolated gallbladder injury due to blunt abdominal trauma is often difficult to diagnose preoperatively because of multiple factors such as being a very rare pathology and the variable and nonspecific clinical manifestations depending on the injury pattern [1,2]. Many cases are diagnosed early using modalities such as ultrasound (US), computed tomography (CT), magnetic resonance imaging (MRI), peritoneal aspiration, endoscopic retrograde cholangiopancreatography (ERCP), scintigraphy, and explorative laparoscopy [3–5]. Searching relevant English literature, we selected 33 published articles with 34 cases of gallbladder injury with at least one of the above diagnostic methods performed. In these studies, the most prominent method was contrast-enhanced CT as a sensitive and effective diagnostic tool for early detection of gallbladder injuries.

## Case report

A 47-year-old male presented with trauma to the right upper quadrant area sustained in a self-falling motorbike accident at 23:00. He was not exposed to COVID-19 because the pandemic was well controlled in Vietnam at that time with only 61 infected cases.

The patient had a history of chronic alcohol consumption and narcotics use. Ten years ago, he was diagnosed with pulmonary tuberculosis and was found to be positive for the human immunodeficiency virus (HIV). The patient presented with abdominal pain that gradually increased and was admitted to the emergency department of our hospital 6 hours after the trauma in a state of drunkenness, complaining of right upper abdomen and lower chest pain. His vital signs such as heart rate, blood pressure, and temperature were unremarkable. On physical examination, he was found to have a pronounced right upper quadrant tenderness, abdominal distention, and no signs of peritoneal irritation. His laboratory findings demonstrated a decreased red blood cell count 2.8 T/L (normal range 4.3-5.7 T/L) and an elevated white blood cell count 18.3 G/L (normal range 3.5-10.5 G/L). Despite of a positive HIV test, no clinical signs of immunosuppression from HIV on the document of patient was found. All other test results were within normal limits.

**Fig. 1:**
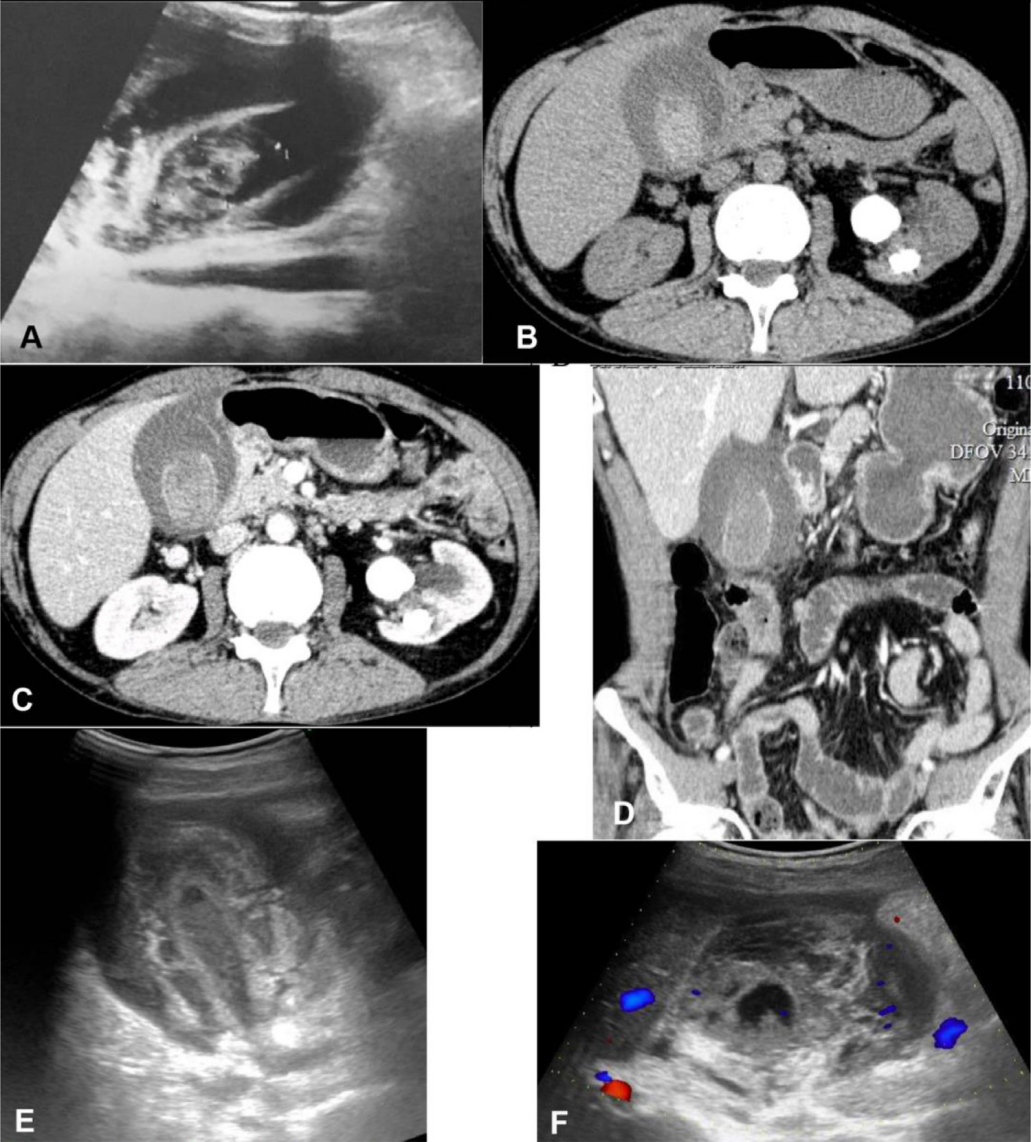
A few hours after hopitalisation, galblladder ultrasound (US) (A) showed a thickned wall, heterogenous material of intraluminal blood clot and pericholecystic fluid; Computed tomography (CT) confirmed a high density of intraluminal bloody clots on non-contrast CT (B) and intramual disection with discontinuity of the enhanced mucosal wall located on the liver bed (C and D). On day 4 post admission, US showed a diffuse gallbladder wall thickening (E) and avascularity on color Doppler (F) surrounded by pericholecystic fluid thickness of 23 mm and an echogenic blood level in the lumen.

Chest radiography revealed fibrotic changes of old tuberculosis in the right upper lobe. On standard abdominal radiography, there were no signs of intestinal perforation or bowel occlusion. However, a fracture of the right lateral 11th rib and a staghorn calculus in the left kidney was noted. Urgent US showed a thickened gallbladder wall with bloody clots in the lumen and a large pericholecystic fluid; there were no signs of liver injury or intraperitoneal free fluid (Fig. 1A). Abdominal CT revealed blood in the lumen and loss of continuity of gallbladder wall mucosa enhancement, suggesting intramural hematoma without active bleeding. No signs of liver injuries or dilation of the biliary ducts was seen (Figs. 1B, C, D). The patient refused surgery and was admitted for conservative treatment. The patient developed bloody stools 2 days later which lasted for a few days. On day 4, US showed a diffuse intramural hematoma and lack of vascularity on color Doppler surrounded by pericholecystic fluid with blood levels in the collapsed lumen (Figs. 1E and F). After 11 days of hospitalization, the abdominal pain was relieved, and the patient was discharged.

**Fig. 2:**
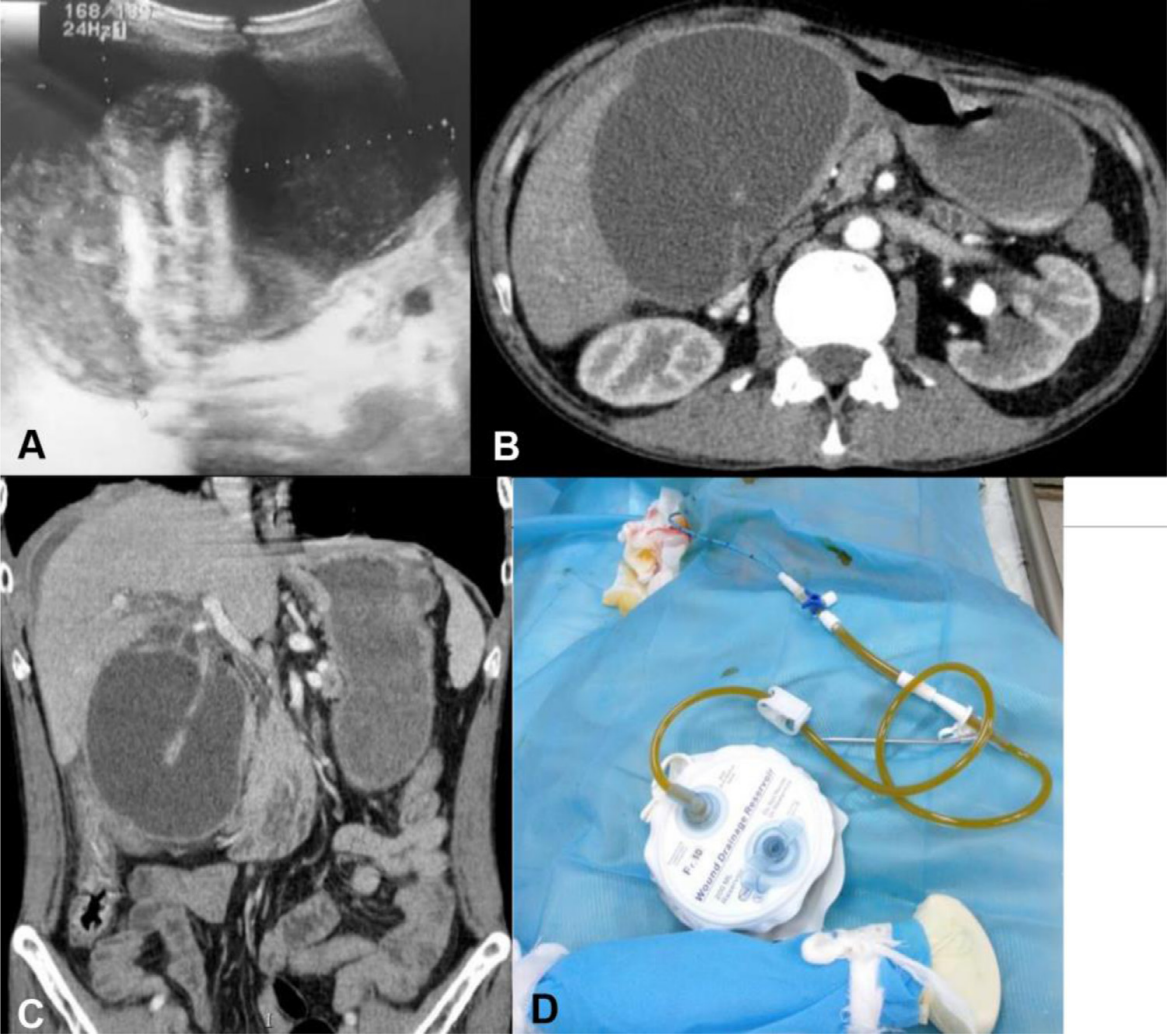
Ultrasound (US) performed on day 17 demonstrated a diffuse gallbladder wall thickness surrounded by a voluminous mass of pericholecystic fluid measuring 9 × 10 × 12 cm (A). Abdominal contrast-enhanced computed tomography (CT) confirmed ultrasound (US) findings and enhancement of a thickened gallbladder wall, free fluid around the liver and the right paracolic gutter was seen (B and C). US-guided percutaneous transhepatic drainage of the gallbladder was performed and around 350 ml of bile fluid was aspirated (D).

On day 17, the patient presented with aggravated pain in the right upper quadrant with nausea, a mild fever of 37.4°C, and an abdominal palpable mass. US revealed an enlarged mass of pericholecystic fluid surrounding the collapsed gallbladder wall (Fig. 2A). Contrast CT showed a voluminous fluid collection surrounding the totally collapsed gallbladder mucosa (Figs. 2B and C). US-guided percutaneous transhepatic drainage of the gallbladder was performed and 350 ml of yellowish fluid without blood was aspirated, suggestive of bile fluid which was confirmed by testing (Fig. 2D). The patient was discharged after 8 days without symptoms with the catheter still in place as he refused surgery.

**Fig. 3:**
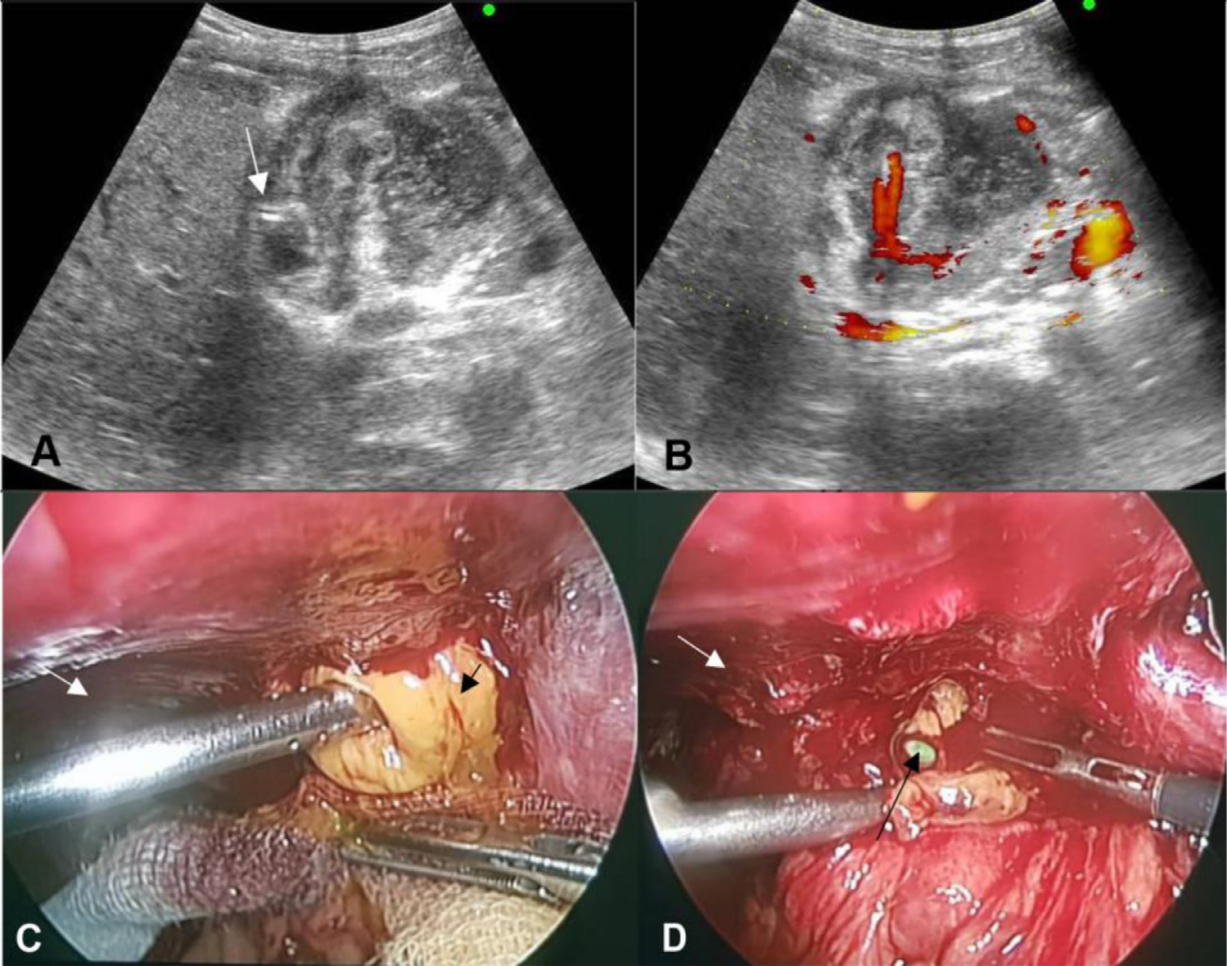
Preoperative ultrasound (US) done on day 50 showed the drain outside the lumen (A), a thickened wall with vascularity on Doppler (B). Laparoscopic view (C) presented a distended dark brown wall mass (white arrow); opening of the wall revealed concentrated yellow purulent fluid (black arrow). After the removal of pus (D) the collapsed gallbladder (white arrow) and catheter drain (black arrow) was seen.

On day 48, the clinical and laboratory findings were not abnormal, and the catheter drained very little fluid but was still purulent. US revealed that the gallbladder area was a solid mass with signs of atrophy and that the tip of the catheter was located in the gallbladder mass. The gallbladder had nearly completely collapsed and the mucosal walls were irregularly thick and showed increased vascularity on color Doppler (Figs. 3A and B). There was no free fluid in the abdomen. After explaining the risks of complications, the patient agreed to surgery. Laparoscopy revealed a 7 × 8 cm gallbladder mass surrounded by the omentum, transverse colon, duodenum, and with adhesions to the abdominal and hepatic walls. The gallbladder mass was found to be concentrated with yellow purulent fluid upon opening (Figs. 3C and D). Cholecystectomy was performed by laparotomy as the cystic duct dissection was difficult due to adhesions to the duodenum. Upon opening the gallbladder postoperatively, the mucosa of the gallbladder was perforated and separated from the liver bed. Pathological examination showed a wall thickness of 5-8 mm, with chronic proliferative inflammation accompanied by necrotic areas. The patient recovered uneventfully and was discharged on the seventh day postoperatively.

## Discussion

### Epidemiology

Gallbladder injuries are uncommon, and most cases occur following penetrating trauma, with an incidence of 1.8%-2% seen in surgery for blunt abdominal trauma [6,7]. Gallbladder injuries are usually associated with other blunt traumatic complications, including liver laceration, duodenal perforation, and splenic laceration [7]; thus, they can be overlooked and usually only detected during surgery. Due to its small size and protection by the surrounding liver, omentum, and the rib cage, isolated gallbladder injuries are rare.

### Predisposing factors

Most traumatic gallbladder injuries arise from compressive and shearing forces following motor vehicle accidents; other causes include injuries from falls, kicks, or blows to the abdomen [7]. Therefore, most cases occur predominantly in men. Several factors predispose patients to gallbladder injuries in blunt abdominal trauma. The long mesentery of the gallbladder and its normally thin walls are more prone to a chronically inflamed thick-walled gallbladder [6], the mobility of gallstones in chronic cholecystitis may cause mucosal tears with intraluminal bleeding [8,9]. A distended gallbladder at the time of trauma or due to chronic alcohol abuse causes an increased tone of the sphincter of Oddi [6,8,10]. In addition, alcohol intoxication may lead to relaxation of the abdominal wall, while meals theoretically increase the likelihood of perforation [11]. The gallbladder is placed on a stiff, cirrhotic liver, which appears to be a risk factor [8,12,13]. Trauma mechanism is by the acute deceleration of a relatively mobile gallbladder due to falling from a great height [4].

### Classification of gallbladder injuries

Blunt gallbladder injuries are commonly classified into 3 types: contusion, avulsion, and laceration/perforation [6,7,11,14]. Contusion causes an intramural hematoma, making it difficult to diagnose preoperatively and may be confirmed on pathology. Avulsion injury of the gallbladder is classified either as a partial or complete detachment from the liver bed or total if the cystic artery and duct are affected [1,10]. Gallbladder perforation is the most common complication that can occur immediately after trauma or progression of traumatic gallbladder contusion. Moreover, transmural perforation can occur in any part of the wall, but the fundus seems to be the most vulnerable area, which is the weakest point in response to direct blows.

### Clinical presentation

Clinical presentations are highly vague with unspecific right hypochondrial pain and can range from an acute abdomen to a slow progression of abdominal symptoms. This can lead to a late diagnosis or delayed surgery several weeks after trauma [6,15,16]. When the gallbladder perforates, bile with/without blood extravasates into the peritoneal cavity leading to bile peritonitis which makes diagnosis difficult and causes a delay in treatment [17]. Subsequently, the ruptured gallbladder wrapped by the omentum forms boundaries with the fibrinous adhesions; therefore, clinical symptoms may not be obvious until the mass enlarges, causing a dull pain and a palpable mass [6], as in our patient. Acute abdomen and shock may also present in patients with delayed gallbladder rupture after blunt abdominal trauma [16]. Depending on the amount of blood loss, mild hemobilia presenting as melena may occur due to the intraluminal bleeding of the gallbladder wall laceration or contusion, as in our patient.

### Radiographic findings

Sonography is the first-line modality for evaluation and rules out serious intra-abdominal injuries. Appreciation and awareness of sonographic findings associated with gallbladder perforation are critical for early diagnosis. These findings include heterogeneous hyperechoic blood inside the gallbladder, ill-defined or thickened gallbladder wall, and distended or collapsed gallbladder with pericholecystic fluid [9,12,13,18]. Experienced examiners may be able to detect gallbladder perforation on US with signs of intraluminal echogenic mass in a hydropic thick-walled gallbladder with a changing hyper-echogenic fluid collection despite initial negative CT scans [9]. Disruption of the gallbladder wall with focal loss of reflectivity may represent perforation [12,14]. Non-visualization of the gallbladder on US should raise the suspicion of a traumatic gallbladder avulsion [10]. However, there are also cases in which there are no abnormal gallbladder findings [4,19].

CT has been shown to be the most effective diagnostic tool for detecting gallbladder injuries; it may help detect the spontaneous density of hematoma in the gallbladder and/or hemoperitoneum [6,8,20,21]. Contrast-enhanced CT findings present many different features and facilitate preoperative diagnosis. Active bleeding, such as intraluminal hemorrhage within the gallbladder lumen [1,13], leakage of contrast from the region of the porta hepatis into the right iliac fossa [18]. Using maximum intensity projections, contrast extravasating from the tear of the cystic artery in the gallbladder wall can be seen [22]. The delayed phase can be useful for differentiating progression of true hemorrhage with an increased amount of dense fluid within the gallbladder from a nontraumatic gallbladder injury where there is a definite fluid–fluid levels that remains stable on the delayed images [5,23]. Discontinuous wall or collapsed gallbladder with pericholecystic and peritoneal fluid usually presents with gallbladder perforation [8,11,12,16,17]. Intramural dissection of the gallbladder wall with subsequent intramural and intraluminal hematoma may progress from a distended gallbladder contusion [4], as in our patient. Non-visualization of the gallbladder may reveal a completely avulsed gallbladder [10]. Low-grade hepatic injuries accompanied by pericholecystic, perihepatic, and free fluid may mask gallbladder rupture [17,19]. Occasionally, CT can only depict peritoneal fluid but may not identify the source [15].

MRI is rarely indicated for abdominal trauma; however, it may be complementary to rule out a hematoma from a gallbladder perforation detected on CT findings along with its ability to depict mural discontinuity of the perforated gallbladder wall [6]. Moreover, MRI can suggest a non-hemorrhagic free fluid in cases of liver injury, with bilious fluid signals with decreased T1W and increased T2W compared to an increase in both T1W and T2W of hemorrhagic fluid in the subacute phase (2 days to months) [24].

### Miscellaneous findings

Laboratory testing may demonstrate altered liver functions due to clot formation and intermittent common bile duct obstruction and may manifest as mild anemia. Elevated bilirubin is frequent due to the absorption of bile from the peritoneum [20].

Diagnostic peritoneal aspiration with/without guiding US can be used in cases of gallbladder perforation to confirm the presence of bile fluid; however, it is nonspecific and may be caused by trauma to the biliary tree, liver, and the upper gastrointestinal tract [7]. This method can help corroborate the imaging findings from a CT scan, suggesting gallbladder perforation [19].

ERCP may not be the first choice as a diagnostic tool. It localizes the source of the presumed biliary injury presenting with bilious fluid aspiration before surgery [19]. ERCP findings show extravasation of contrast from the perforated gallbladder, which helps confirm the diagnosis; however, the main purpose is for a temporary biliary stent placement for conservative treatment [12,25,26].

Biliary isotope scintigraphy is an uncommon modality that is not always available in emergency departments. It can reveal free intra-abdominal leakage of bile when the gallbladder is ruptured, but on its own may not show enough information and should be combined with CT findings [12,18].

Diagnostic laparoscopy can be performed in equivocal trauma cases to identify gallbladder injury in stable patients and, once confirmed, proceed to laparoscopic cholecystectomy [10,18].

### Management

Cholecystectomy is the treatment of choice for gallbladder injuries. Conservative treatment for gallbladder injuries is not recommended and is rarely reported in literature. It seems to be effective in cases of gallbladder contusion with minor tears of the muscularis layer of the gallbladder, causing hematoma within the gallbladder. However, most cases of contusion will undergo cholecystectomy due to the risk of perforation from the intramural hematoma progressing to necrosis in an obstructed hydropic gallbladder due to blood clots [4]. Conservative treatment of gallbladder injuries may be a choice in high-risk candidates for surgery, such as in those on therapeutic heparin for a ventricular thrombus induced by atrial fibrillation [26] and end-stage alcoholic cirrhosis with liver and respiratory failure [12]. In these cases, alternative treatment is usually a temporary biliary stent placement by ERCP with an external peritoneal drain [12,25,26]. An unusual case of perforated gallbladder wall treated by biliary stenting has been published; however, the cause was not mentioned and there was no follow-up information [19]. Cholecystectomy may be deferred and supported by conservative treatment using US-guided percutaneous transhepatic drainage of the percutaneous gallbladder, as in our patient.

## Conclusion

In general, isolated gallbladder injuries are very rare, and preoperative diagnosis is usually difficult, resulting in delayed treatment. Clinical signs suggest acute deceleration of blunt abdominal trauma with manifestation of progressive right hypochondrial pain in patients with chronic alcoholism, narcotic use, or those who are intoxicated. US and CT are the most suitable imaging techniques for confirming the diagnosis. In addition, depending on specific cases, diagnosis can be supplemented by other methods, such as peritoneal aspiration and ERCP. Conservative treatment is not recommended except in cases of minor contusion or non- traumatic cholecystitis, or if the patient's condition does not permit surgery.

## Author contributions

All authors contributed to data analysis, drafting or revising the article, gave final approval of the version to be published, and agree to be accountable for all aspects of the work

## Patient consent

Written informed consent has been obtained from the patient for the publication of this case report and any accompanying photographs. This case report is an incidental finding in the course of clinical work and has no ethical implications.
